# Cross-Cultural Validation of the Polish Version of the ADI-R, Including New Algorithms for Toddlers and Young Preschoolers

**DOI:** 10.1007/s10578-018-00865-2

**Published:** 2019-01-19

**Authors:** Izabela Chojnicka, Ewa Pisula

**Affiliations:** Department of Health and Rehabilitation Psychology, Faculty of Psychology, University of Warsaw, Stawki 5/7, 00-183 Warsaw, Poland

**Keywords:** Autism spectrum disorder, ASD, Autism Diagnostic Interview-Revised, Adaptation, Validation

## Abstract

Autism Diagnostic Interview-Revised (ADI-R) is one of the most widely used standardized diagnostic instruments for autism spectrum disorder (ASD). This article presents findings from the validation of the Polish version of the Autism Diagnostic Interview-Revised (ADI-R-PL), including new algorithms for toddlers and preschoolers. The validation group consisted of 125 participants: 65 with Autism Spectrum Disorder (ASD group) and 60 in the control group, including individuals with non-ASD disorders and typical development. The normalization group consisted of 178 participants, including 118 with ASD. The ADI-R-PL was found to have good psychometric properties. Confirmatory factor analysis supported both a bifactor structure and three-factor model. The study has generated preliminary information about the psychometric properties of the new algorithms for toddlers and young preschoolers. To the best of our knowledge, this paper is the first to propose new cutoffs in three ADI-R domains for a non-English-speaking population.

## Background

Autism spectrum disorder (ASD) is a heterogeneous neurodevelopmental disorder whose prevalence has risen significantly in recent years, currently reaching or exceeding 1% worldwide [1, 2]. The disorder is defined by its clinical symptoms affecting social communication and manifests in repetitive, restricted patterns of behavior, activity, and interests [3]. The clinical judgment of experienced clinicians informed by standardized diagnostic instruments is the best predictor of stable and reliable ASD diagnoses [4].

In Poland, as in other countries in Central and Eastern Europe, the availability of validated instruments for diagnosing ASD remains problematic [5, 6]. This is a major obstacle for the development of diagnostic standards, although many facilities aim to follow the best practice guidelines of a comprehensive diagnostic evaluation for autism [7]. Professionals tend to use nonstandardized protocols of parent/caregiver interviews and unstructured observation with undetermined psychometric properties [8]. The situation has recently improved with the emergence of validated Polish versions of two instruments with diagnostic application: the Autism Diagnostic Observation Schedule, Second Edition (ADOS-2, [9–11]) and Autism Spectrum Rating Scale [12, 13]. Currently, these are the only measures helpful in the clinical diagnosis of ASD with verified psychometric properties available in Polish.

This paper presents findings from the validation of the Polish version of the Autism Diagnostic Interview-Revised (ADI-R, [14]), a standardized parent/caregiver ASD diagnostic interview protocol. This measure, along with the ADOS-2, is the golden diagnostic standard in many countries. In recent years there have been attempts made to adapt it for assessing children of less than 24 months of age [15–17]. This area of this instrument’s application is still under-explored and merits more attention due to the possibility of early detection of ASD.

### Description of the ADI-R

The ADI-R provides information on the history of development and current functioning of the assessed individual. The interview covers a wide range of information, including family status, education, treatment and therapy history, early development and the first concerning symptoms, as well as developmental milestones and any skill regression. Interviewees are also asked about behavioral problems, sensory processing abnormalities, the presence of special isolated abilities, and other issues associated with difficulties seen in ASD. The instrument has application both in the diagnostic process and in intervention planning and efficacy assessment [e.g., 18]. It is also widely used in scientific research to verify the validity of participant selection in ASD groups [e.g., 19, 20]. In a research setting, information gathered in the interview is evaluated by two independent and appropriately trained raters.

The presence of ASD symptoms is determined by two ADI-R diagnostic algorithms. One of them is designed for children aged 2 years to 3 years, 11 months, and the other for individuals 4 years and older. The items attributed to the algorithms make up three domains: (1) Qualitative Abnormalities in Reciprocal Social Interaction; (2) Qualitative Abnormalities in Communication; and (3) Restricted, Repetitive, and Stereotyped Patterns of Behavior. Also taken into account is the time symptoms first manifested in the child (fourth domain). The results obtained in each domain are then compared with cutoff points established by testing large groups of individuals with ASD and control groups [14]. The final determination in ADI-R using a diagnostic algorithm that takes into account development history falls into one of two categories: autism versus nonspectrum. Additionally, three Current Behavior Algorithms focusing on present functioning and used for treatment and educational planning can also be employed. Key information about the structure of the ADI-R and score calculation is summarized in Table 1.

**Table 1:** Structure of the ADI-R interview booklet and algorithms

**Interview protocol**	
A comprehensive interview form composed of 93 items is used. A diagnostician scores most items in the interview by selecting one of the codes: 0, 1, 2, 3, 7, 8, 9. The codes are defined as follows: no definite behavior of the type specified (score of 0); behavior of the type specified probably present but defining criteria not fully met (score of 1); and definite abnormal behavior of the type described in the definition and coding (score of 2), with a score of 3 used to indicate extreme severity. The other scores mean: definite abnormality in the general area of the coding, but not of the type specified (score of 7); not applicable (score of 8); and not known or not asked (score of 9)	
**Algorithms for children aged 2 years or older**	
Composed of five age-specific algorithms	Two Diagnostic Algorithms based on developmental history and used for diagnostic purposes Three Current Behavior Algorithms focusing on present functioning and used for treatment and educational planning
An examiner converts the item codes to algorithm scores Ratings of 3 to algorithm scores of 2 Ratings of 7, 8, 9 to algorithm scores of 0 The examiner does not convert ratings of 0, 1, or 2	
The examiner transfers ratings of 0, 1, and 2 directly to the algorithm scores into four domains Qualitative Abnormalities in Reciprocal Social Interaction Qualitative Abnormalities in Communication Restricted, Repetitive, and Stereotyped Patterns of Behavior Abnormality of development evident at or before 36 months	
The scores in each domain are added up. Scores equal to or higher than the cutoff in each of the four domains indicate the determination of *autism* in ADI-R	

Based on Rutter et al. [14]

Research conducted in the United States and Great Britain has shown that ADI-R possesses high interrater reliability and test–retest reliability [14, 21, 22], as well as high diagnostic validity, correctly differentiating between children with autism and children with intellectual disability or language impairment [14]. Good validity of the ADI-R has been confirmed in various age groups: both preschoolers [23] and adolescents [24], and in people with various levels of intelligence.

### New Algorithms for Toddlers and Young Preschoolers

In order to expand the application of the ADI-R and make it a useful instrument for diagnosing small children, new algorithms have been developed for toddlers and young preschoolers from 12 to 47 months of age [15–17]. The algorithms are age- and speech-development-appropriate: algorithm 12–20/NV21–47 for children from 12 to 20 months of age and nonverbal children from 21 to 47 months of age; algorithm SW21–47 for children with single words from 21 to 47 months of age; and algorithm PH21–47 for children with phrase speech from 21 to 47 months of age. Due to the children’s age the final score is interpreted not in terms of a diagnostic classification, but as three-point ranges of concern: Little-to-No Concern, Mild-to-Moderate Concern, and Moderate-to-Severe Concern. The properties of the new algorithms are presented in Table 2.

**Table 2:** Structure of the ADI-R new algorithms for toddlers and young preschoolers

**Algorithms for toddlers and young preschoolers from 12 to 47 months of age**			
Composed of three algorithms		12–20/NV21–47 for children from 12 to 20 months of age and nonverbal children from 21 to 47 months of age SW21–47 for children with single words from 21 to 47 months of age PH21–47 for children with phrase speech from 21 to 47 months of age	
The choice of algorithm depends on the child’s age and scores on the ADI-R item “Overall Level of Language” Score “0” → algorithm PH21–47 Score “1” → algorithm SW21–47 Scores “2” or “3” → algorithm 12–20/NV21–47 An examiner converts item codes to algorithm scores ratings of 3 to algorithm scores of 2 ratings of 7, 8, 9 to algorithm scores of 0 The examiner does not convert ratings of 0, 1, or 2			
The examiner transfers ratings of 0, 1, and 2 directly to the algorithm scores into: two domains (algorithms 12–20/NV21–47 and SW21–47): Social Affect (SA) and Repetitive and Restricted Behaviors (RRBs), or three domains (algorithm PH21–47): Social Communication (SC), RRBs and Reciprocal Peer Interaction (RPI)	For algorithms 12–20/NV21–47 and SW21–47 SA Total + RRB Total = Total algorithm score		Results are converted to the three-point ranges of concern to be used for clinical purposes. Scale with two cutoffs has been provided
	For algorithm PH21–47 SC Total + RRB Total + RPI Total = Total algorithm score		

Based on Kim and Lord [16]

Empirical findings suggest that the new algorithms for younger children demonstrate higher sensitivity and specificity than the original algorithm [16]. The one exception is the measure of sensitivity in the case of children aged 12–20 months and nonverbal children aged 21–47 months: sensitivity was higher in the previous Current Behavior Algorithm.

### Psychometric Properties of Non-English Versions of the ADI-R

The preponderance of research on the psychometric properties of the ADI-R was conducted on Englishspeaking populations [25, 26]. There is less information about the properties of translations of the ADI-R and their usefulness in diagnosing ASD in non-English speaking populations.

One of the first studies of that kind evaluating the German language version of the ADI-R was conducted on 22 individuals with autism [27]. It found that it had good interrater reliability, with the intraclass correlation coefficients slightly lower than in the original, which the authors explained by the high homogeneity of the study sample. Good diagnostic validity of the German version of the ADI-R was confirmed by Mildenberger et al. [28] in a study on children with autism and children with a specific receptive language disorder. Similarly, the Bulgarian translation of the ADI-R possessed high reliability, as measured by the test–retest method [29]. This version, like the German one, demonstrated slightly lower interrater reliability than the original, possibly due to the fact that of the pair of raters who evaluated information obtained in the interview, only one individual had completed official training in the use of the ADI-R. The Japanese version of the ADI-R [30] was also characterized by high reliability (verified on a group of 51 individuals) and validity (group of 317 individuals). It had high interrater reliability and intraclass correlation coefficients (greater than 0.80 for all three domains of the ADI-R). Its diagnostic validity was also high (sensitivity and specificity of 0.92 and 0.89, respectively).

The usefulness of the ADI-R at distinguishing ASD from other disorders was also evaluated in a large study of over 1,200 Dutch children [31]. It found that the specificity of diagnosis increased significantly when the ADOS results were taken into account. A similar conclusion was reached by Zander et al. [32] in their research on the diagnostic validity of the new ADI-R algorithms in a Swedish sample of toddlers and young preschoolers. High correlation of the autism classification determined using the ADI-R with clinical diagnosis and classification using the ADOS was also demonstrated for the Greek translation of the ADI-R [33], and between the ADI-R and clinical diagnosis for the Finnish form of the instrument [34].

The new ADI-R algorithms have also been tested empirically on a non-US sample [15]. Specificities for the clinical and research cutoffs resembled the ones in the US studies, except in the SW21–47 cell, which had a lower specificity on the clinical cutoff (0.70). However, the sensitivities in the non-US sample were lower for all developmental cells compared to the original Kim and Lord study [16].

Thus, as evidenced by the studies mentioned above, various language versions of the ADI-R proved useful in diagnosing ASD in a number of countries. This paper presents the reliability and validity of the Polish version of the ADI-R, including the psychometric properties of the new algorithms for toddlers and young preschoolers. As such, it provides novel information about a non-English version of the ADI-R and, to the best of our knowledge, is one of the first projects of its type in Central and Eastern Europe (following the Bulgarian version, [29]).

## Methods

### Participants

The study of reliability and validity of the ADI-R-PL included 125 individuals: 65 with clinical diagnosis of ASD (hereinafter the ASD group) and 60 referred to collectively as the control group, with nonspectrum disorders (*n* = 18) and typical development (*n* = 42). The normalization group was made up of 178 individuals, including 118 with ASD and 60 controls.

Inclusion criteria for participants were (1) age ≥ 24 months, (2) clinical diagnosis of autism spectrum disorder determined by a psychiatrist based on ICD-10 diagnostic criteria [35] or another diagnosis in the case of participants with nonspectrum disorders, and (3) in the control group consisting of typically developing individuals, no diagnosed developmental disorders, neurological, or psychiatric conditions or suspected developmental problems. Due to the use of the ADOS-2 [9, 10] in the study, the exclusion criteria additionally included severe hearing, sight, and mobility impairments. All verbal participants in the study and all interviewed parents/caregivers were Polish speakers.

The ratio of females to males in the ASD group was 1: 4.42. For the purposes of the normalization analysis, 53 individuals with ASD were added to this group. The ratio of females to males in the whole normalization sample was 1: 4.88. The higher ratio of males in the ASD group reflects the gender disproportion in the population of people on the autism spectrum [1].

The group with nonspectrum disorders consisted mostly of individuals with intellectual disability or speech disorders (each impairment accounting for approximately half of the group). The demographic profile of the validation sample is shown in Table 3.

**Table 3:** Demographics of the sample

	ASD group	Control group	*p*
*N* validation sample (% of females)	65 (18.46)	60 (43.33)	
*N* normalization sample (% of females)	118 (17.8)	60 (43.33)	
*n* of verbal participants in the normalization sample (score 0 the ADI-R item “Overall Level of Language”)	84	55	
*n* of participants with limited speech in the normalization sample^a^	34	5	
Gender (F:M)	1:4.42	1:1.4	
Chronological age in years *M* (*SD*)	9.18 (5.68)	10.44 (8.52)	0.415
Age range in years	2.5–21.5	2.5–39.0	
IQ scores^b^			
Leiter Scale score (*n* = 52) *M (SD)*	94.76 (27.93)	106.86 (27.13)	0.070
WISC-R (*n* = 34) IQ *M (SD)*	87.94 (27.68)	102.63 (31.40)	0.262
WAIS-R (*n* = 25) IQ *M (SD)*	99.83 (41.59)	107.15 (46.74)	0.326
The characteristics of the new algorithms for toddlers and young preschoolers group			
Gender (F:M)	1:5	1.13:1	
Chronological age in months *M* (*SD*)	39.25 (6.36)	39.35 (4.94)	0.985
Age range in months	30–47	30–47	
Leiter Scale IQ *M (SD)*	84.55 (23.28)	113.18 (21.57)	0.049
ADI-R-PL scores from the Algorithms for children aged 2 years or older *M (SD)*			
Qualitative Abnormalities in Reciprocal Social Interaction	20.37 (7.36)	4.22 (5.94)	< 0.001
Qualitative Abnormalities in Communication	13.92 (5.28)	3.57 (4.60)	< 0.001
Restricted, Repetitive, and Stereotyped Patterns of Behavior	7.74 (3.91)	1.35 (1.98)	< 0.001
ADI-R-PL scores from the new algorithms for toddlers and young preschoolers *M (SD)*			
Social affect (SW21–47)	9.75 (6.15)	1.59 (2.45)	0.001
Repetitive and restricted behaviors (SW21–47)	6.33 (4.64)	1.82 (2.24)	0.001
Total Algorithm Score (SW21–47)	16.08 (9.66)	3.41 (3.57)	0.001
Social communication (PH21–47)	6.00 (5.79)	1.14 (1.99)	0.017
Repetitive and restricted behaviors (PH21–47)	5.60 (2.30)	1.21 (1.12)	0.003
Reciprocal and peer interactions (PH21–47)	1.80 (1.48)	0.71 (1.20)	0.086
Total Algorithm Score (PH21–47)	13.40 (7.09)	3.07 (3.47)	0.009

*ASD* autism spectrum disorder, *M* Mean, *SD* standard deviation, *WISC-R* the Wechsler Intelligence Scale for Children-Revised, *WAIS-R* The Wechsler Adult Intelligence Scale, Revised, *SW21–47* algorithm for children with single words from 21 to 47 months of age, *PH21–47* algorithm for children with phrase speech from 21 to 47 months of age

^a^Scores 1 and 2 on the ADI-R item “Overall Level of Language”;

^b^Participants under the age of 3 (*n* = 9) were assessed using the Polish Child Development Scale with percentile scores representing the level of global development instead of IQ scores; In five cases information regarding cognitive level was missing

The majority of interviewees were mothers (82.4%). In only 4.8% of cases the information was provided by fathers, and in the remaining cases both parents were interviewed. Of the respondents, 55.2% had higher education, 24% had secondary education, and 8% had primary or vocational education. Participants living in cities of at least 100,000 residents made up 73.2%, towns up to 100,000 inhabitants 21.1%, and villages 5.7%.

### Measures and Procedure

#### Adaptation of the Polish Version of the ADI-R

The first stage in the development of the Polish version of the ADI-R (ADI-R-PL) was the translation of the original (Western Psychological Services Edition) to Polish by the present author (xx) in collaboration with a professional translator. Secondly, the translation was proofread by a native Polish linguist, followed by correction and revision. The next step was a blind back translation by an independent translation company, which was then checked by Professor Ann Le Couteur, one of the instrument’s original authors. Following revisions recommended by the author, the Polish version of the ADI-R was approved by the publisher, Western Psychological Services, for use in scientific research.

The Polish version of the ADI-R preserves the structural equivalence in terms of the graphical layout of the protocol, text, and item formatting. The translation is true to the original in terms of item content. The grammatical structure of questions, difficulty of terms used, and lexical similarity have also been preserved. The English nursery rhymes were replaced by their Polish equivalents (the social games “Here We Go’Round the Mulberry Bush” and “Ring A Ring O’Roses” were replaced by the Polish games: “Kółko graniaste”, “Stary niedźwiedź mocno śpi” and “Baloniku nasz malutki”).

#### Other Measures

##### Autism Diagnostic Observation Schedule, Second Edition

As regards convergent validity analysis, 93.6% of participants (*N* = 117) were tested using the Polish version of the ADOS-2-PL [11]. The ADOS-2 is a standardized, semistructured observation schedule for diagnosing individuals on the autism spectrum [9, 10]. It comprises five assessment modules for assessing individuals of different ages and language development levels, from children of a chronological and mental age of at least 12 months to adults. The ADOS-2-PL features high reliability and validity.

##### Social Communication Questionnaire

In order to further assess convergent validity we used the author-reviewed Polish version of the Social Communication Questionnaire (SCQ), accepted for research use by the Western Psychological Services, the copyright holder. The SCQ is a screening questionnaire for ASD based on the Autism Diagnostic Interview-Revised. It consists of 40 yes-or-no items concerning communication skills and social functioning. It can be useful for screening purposes in children over 4.0 years with a mental age over 2.0 years who may have ASDs [36]. The Polish version of the SCQ is characterized by having good psychometric properties (Pisula et al. 2018, unpublished manuscript).

##### IQ Measurement

The intellectual functioning of all participants was also tested. Several measures were used due to variance in age, language skills, and intellectual development, namely: (a) the Leiter International Performance Scale [37] for nonverbal participants aged 3.0–15.11; (b) the Wechsler Intelligence Scale for Children-Revised [38] for verbal children and adolescents aged 6.0–16.11; and (c) the Wechsler Adult Intelligence Scale [39] for verbal participants older than 16.11. Children younger than 36 months were assessed using the Polish Child Development Scale (Dziecięca Skala Rozwojowa, DSR, [40]).

### Design and Analysis

The project was approved by the Faculty of Psychology, University of Warsaw Research Ethics Committee. Informed consent was signed prior to participation in the study by: (a) the parents of participating children under 16 years of age, (b) the parents of participating children aged 16 and older and participants themselves (in relation to the ADOS-2 and IQ test). By approval of the Ethics Committee, the interviews were video-recorded with the consent of participants and/or their parents or caregivers.

The ADI-R-PL was conducted as part of a research evaluation. Recruitment was conducted in four cities in Poland. Participants were contacted through diagnostic and therapeutic centers specialized in diagnosing autism spectrum and other disorders, as well as foundations and associations supporting individuals with developmental disabilities, nurseries, kindergartens, and public schools. The aim was to enroll individuals who were diagnosed with childhood autism, Asperger syndrome, or pervasive developmental disorders unspecified within one year of the start of the study. In most cases where the time from diagnosis was longer, psychiatrists working on the project assessed the current functioning of participants using the ICD-10 diagnostic criteria [35].

Each ADI-R-PL protocol, along with the relevant diagnostic algorithm, was completed independently by two professionals who met the research requirements of standardized administration and scoring reliability. The assessment of almost half of the participants (49%) was carried out in real time and independently by two diagnosticians, while in the remaining cases the second professional made his assessment based on a video recording.

Fifty-one participants were reassessed in order to estimate the stability of the ADI-R-PL scores. The two assessments were made from 1 to 14 months apart (mean 5.37 months; the gap was shortest with the youngest participants—those aged 8 years or less—and the longest for adults).

The psychometric properties of the algorithms for toddlers and young preschoolers from 12 to 47 months of age were calculated on the basis of scores from 29 children aged 30–47 months, with the SW21–47 algorithm applied in 10 cases and the PH21–47 in 19 cases. The psychometric properties of the 12–20/NV21–47 algorithm were not evaluated due to the inclusion criterion of at least 24 months of age at the time of enrollment (as recommended for the ADI-R according to Rutter et al. [14]).

In the case of the new algorithms, due to the small number of participants aged 30–47 months the analysis was limited to reliability (interrater reliability, test–retest reliability, and internal consistency) and between-groups comparisons (Mann–Whitney’s U test). Sensitivity and specificity analyses were not conducted due to sample limitations.

Analyses performed for the algorithms for children aged 2 years or older involved determining the factor structure; diagnostic validity estimated via sensitivity, specificity, and positive predictive value; and convergent validity assessed by comparing the results in the Polish version of the ADI-R with scores in other measures used to diagnose ASD: ADOS-2 and SCQ.

The reliability of algorithms for children aged 2 years or older was estimated using three methods: interrater reliability (percent agreement, weighted kappas, and intraclass correlation coefficients), test–retest reliability (intraclass correlations), and internal consistency reliability (by calculating Cronbach’s alpha). Computations were made using the IBM SPSS Statistics 17.0 suite [41]. For reliability analyses, scores of 7, 8, and 9 were converted to zeros, while scores of 3 were recoded to 2, as they appear on the algorithms.

Analyses performed for the algorithms for children aged 2 years or older involved determining the factor structure; diagnostic validity estimated via sensitivity, specificity, and positive predictive value; and convergent validity assessed by comparing the results in the Polish version of the ADI-R with scores in other measures used to diagnose ASD: ADOS-2 and SCQ.

Confirmatory Factor Analysis (CFA) was conducted using the maximum likelihood method. In accordance with the procedure proscribed by the authors of the ADI-R, codes 7, 8, and 9 were recoded to 0, while code 3 was recoded to 2 [14]. Estimation of the fit of Polish data to the original three-factor model (including factors (A) Qualitative Abnormalities in Reciprocal Social Interaction, (B) Qualitative Abnormalities in Communication and (C) Restricted, Repetitive, and Stereotyped Patterns of Behavior) and to the DSM-5/ICD-11 two-factor model (social communication and stereotyped behavior factors) was based on the CFIs (comparative fit index) and RMSEA values (root-mean-square error of approximation). Calculations were made using the SPSS Amos 17.0 suite [42].

The comparison of the scores of the pooled group of participants diagnosed with ASD and those obtained by the control group was carried out using Student’s *t* test for independent samples.

ROC (Receiving Operating Characteristics) curves were used in the analysis of sensitivity and specificity. The codes 7, 8, and 9 were recoded to 0 and code 3 was recoded to 2 for the purposes of this analysis as well.

The agreement between the ADI-R-PL results with clinical diagnosis, ADOS-2, and SCQ diagnosis was calculated pairwise, using Cohen’s kappa (κ) coefficients. In addition, logit regression analysis was conducted to determine the effect of age and sex on the agreement between the ADI-R-PL and clinical diagnosis.

## Results

### Reliability

Kappas at or above 0.75 were considered excellent, κ = 0.60–.74 were considered satisfactory, κ = 0.40–.59 were considered moderate, and kappas below 0.40 were considered fair [43]. All intraclass correlation coefficients (ICCs) for interrater agreement for the algorithm for children aged 2 years or older as well as the algorithms for toddlers and young preschoolers were excellent (0.96–1.00, Table 4).

**Table 4:** Intraclass correlations for interrater and test–retest reliability and Cronbach’s alphas for internal consistency analysis of the ADI-R-PL

Domain	Interrater reliability	Test–retest reliability	Internal consistency
Algorithms for children aged 2 years or older			
Qualitative Abnormalities in Reciprocal Social Interaction	0.99	0.91	0.95
Qualitative Abnormalities in Communication	0.99	0.88	0.89
Restricted, Repetitive, and Stereotyped Patterns of Behavior	0.98	0.90	0.85
Algorithms for toddlers and young preschoolers			
Social affect (SW21–47)	0.98	0.62	0.92
Repetitive and restricted behaviors (SW21–47)	0.96	0.91	0.90
Total Algorithm Score (SW21–47)	0.97	0.83	0.95
Social communication (PH21–47)	1.00	0.63	0.89
Repetitive and restricted behaviors (PH21–47)	0.98	0.79	0.63
Reciprocal and peer interactions (PH21–47)	1.00	0.74	0.64
Total Algorithm Score (PH21–47)	1.00	0.55	0.90

*SW21–47* algorithm for children with single words from 21 to 47 months of age, *PH21–47* algorithm for children with phrase speech from 21 to 47 months of age

Excellent test–retest reliability values (ICCs) were obtained in the case of domains from the algorithms for children aged 2 years or older (0.88–0.91). As for the domains from the algorithms for toddlers, excellent ICCs were obtained for RRBs (0.91) and Total Score (0.83) for the SW21–47 algorithm, satisfactory for the SA, SC, RRBs, and RPI domains (0.62–0.79), and moderate for Total Score in the PH21–47 algorithm (0.55, Table 4).

Cronbach’s alphas, the internal consistency coefficients, were high [44] for all domains (0.85–0.95) except for RPI (0.64) and RRBs (0.63) in the PH21–47 algorithm (Table 4).

### Factor Structure Analysis

The CFI values for the Polish version of ADI-R was 0.88 and 0.89 for two- and three-factor models respectively, with RMSEA at 0.08 for both models. This means that CFI indicated a slightly lower than acceptable fit to both models, while the RMSEA value was right on the threshold.

Based on those results we decided to retain the original diagnostic algorithms in the ADI-R-PL. This means that the algorithms in the ADI-R-PL consisted of the same items as the algorithms in the original version. Scores in the Polish version were also calculated the same way as in the original. In turn, the cutoff values were changed in three out of four domains of diagnostic algorithms from the Algorithms for children aged 2 years or older, determined on the basis of the scores in the Polish normalization sample (see below).

### Between-Groups Comparisons

Table 3 shows the mean values obtained in individual domains of the diagnostic algorithm by participants on the autism spectrum and by controls. Significantly higher differences emerged in all domains, items, and scales of the ADI-R-PL algorithms for children aged 2 years or older.

As for the algorithms for toddlers and young preschoolers, significantly higher results in the ASD group compared to the control group were obtained in all domains (*p* < .001 and *p* < .01), except for Reciprocal and Peer Interactions (*p* = .086).

### Pair-Wise Agreement Between the ADI-R-PL and ADOS-2 and SCQ Scores

The κ values were 0.64 for the comparison with the ADOS-2 results and 0.73 in the case of comparison with the SCQ. Thus, the diagnostic consistency of the ADI-R-PL with other measures was satisfactory.

### Sex and Age, and Agreement Between the ADI-R-PL and Clinical Diagnosis

The relationships between the sex and age of participants and the agreement of the ADI-R-PL results with the clinical diagnosis was assessed using logit regression. The correlations for the participants’ age were not significant (*p* = 0.62). By contrast, statistically significant relationships were found between the agreement of clinical diagnosis, ADI-R-PL results, and the sex of participants (*B* = 1.03, Exp(B) = 2.81, Wald = 5.77, *df* = 1, *p* = 0.016). Based on Cox and Snell’s *R*^2^ coefficient, the sex factor sex explained 5.0% of variance in results with respect to the agreement between the ADI-R-PL results and clinical diagnosis.

### Sensitivity and Specificity of the Polish Version of the ADI-R

The sensitivity and specificity of the ADI-R-PL were calculated for the Algorithms for children aged 2 years or older by comparing the autism spectrum group with the control group (comprised of typically developing individuals and people with nonspectrum disorders). Cutoffs for individual ADI-R-PL algorithm domains were determined taking into account sensitivity and specificity, as well as positive and negative predictive value coefficients (Table 5). In the case of the Qualitative Abnormalities in Communication scale in the original version of the ADI-R diagnostic algorithms, the following two cutoff points were taken into account: 8 for verbal participants (pursuant to the assessment on the “Overall Level of Language” item) and 7 for nonverbal participants [14]. In the Polish normalization sample, the nonverbal group was small (*n* = 39, including only five subjects in the control group). Findings suggested that a very low cutoff (i.e. 4) had to be specified for this scale, one much lower than the cutoff in the original version of the instrument, whereas for the verbal subjects, the cutoff with best overall accuracy was equal to 7. Therefore, taking into account the size of the sample and original cutoff points, the decision was made to specify one cutoff point with the greatest accuracy (i.e. 7, Table 5) for all participants combined (*N* = 178) equal to the cutoff point for nonverbal participants in the original ADI-R algorithm.

As for the Abnormality of Development Evident at or Before 36 Months domain, the cutoff of 1 was retained, the same as in the original version of the ADI-R. Taking into account the new cutoffs, the sensitivity of the ADI-R-PL was 85%, while the specificity was 93% when comparing ASD versus the whole control group (nonspectrum disorders and typical development) and 78% when comparing ASD versus nonspectrum disorders.

**Table 5:** The sensitivity and specificity of the ADI-R-PL (comparison between the group of individuals with autism and other spectrum disorders with the control group of typically developing individuals and participants with nonspectrum disorders)

Scale (cutoff in the ADI-R)	ADI-R-PL cutoff	Sensitivity	Specificity	PPV	NPV	ROC curve AUC (95% CI)
Algorithms for children aged 2 years or older						
Qualitative Abnormalities in Reciprocal Social Interaction (10)	9	87	87	93	78	0.93 (0.89–.97)
Qualitative Abnormalities in Communication (8 for verbal participants, 7 for participants with limited speech)	7	86	87	91	80	0.89 (0.83–.96)
Restricted, Repetitive, and Stereotyped Patterns of Behavior (3)	3	94	85	93	88	0.98 (0.93–1.00)
Algorithms for toddlers and young preschoolers						
SW21–47						0.91 (0.80–1.00)
Research cutoff	13	67	100	100	81	
Clinical cutoff	8	92	88	85	94	
PH21–47						0.90 (0.79–1.00)
Research cutoff	16	40	100	100	82	
Clinical cutoff	13	40	93	67	81	

*PPV* positive predictive value, *NPV* negative predictive value, *ROC* receiver operating characteristic, *AUC* area under the curve, *CI* confidence interval, *SW21–47* algorithm for children with single words from 21 to 47 months of age, *PH21–47* algorithm for children with phrase speech from 21 to 47 months of age

## Discussion

In the present study, we reported the psychometric properties of the Polish version of the Autism Diagnostic Interview-Revised. In the first part of the discussion we shall focus on the original ADI-R diagnostic algorithms. The algorithms for toddlers and young preschoolers (Kim and Lord 2012b), which have been relatively under-researched so far [15–17], were also analyzed and are discussed separately.

### ADI-R-PL Reliability

In order to assess the reliability of the ADI-R-PL, interrater reliability coefficients were calculated for each domain of the instrument. All kappa values were above 0.90, indicating excellent reliability of the Polish version since they were close to the figures established for the original instrument [45]. The stability of the ADI-R-PL results was also excellent. Our results were slightly lower than those obtained by Lord et al. [45], but higher than in the studies by Hill et al. [29] and Poustka et al. [27]. Internal consistency of the ADI-R-PL diagnostic algorithms, similar to the one reported by de Bildt et al. [31], was satisfactory.

Overall, the obtained results are consistent with the published literature [22, 29, 30, 45]. Based on the strength of the results it can be concluded that the ADI-R-PL is characterized by high reliability, making it a suitable instrument for individual diagnostics for clinical purposes.

### ADI-R Validity

In the confirmatory analysis conducted for the ADI-R-PL, the RMSEA values were at the threshold (0.08), while the CFI values (0.88 and 0.89 for two- and three-factor models respectively) were slightly below the level required to be an acceptable fit (≥ 0.90 [46]) for both models. The CFA conducted by van Lang et al. [47] on a sample of individuals with pervasive developmental disorders (PDD) and social/communication problems using 12 subscale summary scores from the Dutch version of the ADI-R confirmed the three-factor structure, identifying such factors as Impaired social communication, Impaired make-believe and play, and Stereotyped language and Behavior. In another study conducted using a sample of 1,170 verbal children and adults from the Autism Genetic Resource Exchange (AGRE) database, CFA supported a two-factor model, the two factors being Social interaction and Stereotyped speech and restricted/repetitive Behaviors [48]. However, only two studies have examined the factor structure of the ADI-R algorithm items, as was done in the present research. In the work by Lecavalier et al. [49], exploratory factor analysis indicated a three-factor solution similar to the original algorithm. However, unlike the algorithm, the items relating to nonverbal communication loaded on the Social factor. In turn, CFA findings by Snow, Lecavalier, and Houts [50] indicated that the fit indices for the two- and three-factor models were similar and better than the one-factor solution.

Due to the theoretical framework underlying the construction of the ADI-R [14], the compatibility of the instrument with the ICD-10 diagnostic criteria for autism and related disorders [35], and the good psychometric properties of the original ADI-R algorithms documented by multiple authors, including studies in non-English-speaking populations [e.g., 31], ultimately the decision was made to preserve the original structure of the instrument and its individual algorithms. Another argument was the small size of the analyzed sample and its high differentiation in terms of age and other characteristics, although it should be noted that it was still larger than in the research conducted using some other language versions of the ADI-R, e.g. the German [27], Greek [33], and Finnish [34] versions.

Between-groups comparisons revealed statistically significant differences between the ASD and control groups in all scales, subscales, and items making up the ADI-R-PL algorithms, indicating solid discriminant validity of the instrument. Good diagnostic validity was evidenced by high sensitivity (0.86–0.94), specificity (0.85–0.87), and positive predictive values (0.91–0.93) of the ADI-R-PL domains. The AUC values (0.89–0.98) indicate good to excellent accuracy ADI-R-PL accuracy, comparable to the Dutch adaptation (0.77–0.83, [31]). The above parameters were calculated taking into account the newly determined cutoff points. It is a standard procedure in cross-cultural adaptation of assessment measures to develop norms on the basis of data collected in a given population [e.g., 51]. The differences in cut-off values in the present study compared to the original version of the ADI-R, although small, confirm the validity of that approach. The use of norms published for the original versions of instruments carries a high risk of inadequate assessment of symptoms of disorders and may lead to mistaken diagnoses. Our research, as far as we know, is the first to propose cutoffs for three ADI-R domains modified from the original, based on empirical data.

The sensitivity and specificity coefficients and the positive and negative predictive values indicate high agreement of the ADI-R-PL results with clinical diagnosis. We found an interesting correlation between the convergence of the ADI-R-PL and clinical diagnosis with participants’ sex, while no such relationship was found for age. Diagnosis agreement was excellent regardless of sex in the control group and good in the ASD group. Although existing studies on sex differences in ASD are still limited, they suggest a presence of gender differences in the autism spectrum disorder and problems diagnosing ASD in females (see for review: [52]). For example, Dean et al. [53] have suggested that the female social landscape masks social impairments of girls with ASD whereas the male landscape makes it easier to detect the social challenges of boys with autism spectrum disorder.

The convergent validity of the ADI-R-PL was established by comparing ADI-R-PL results with the ADOS-2 [9, 10] and SCQ [36] scores. The agreement was satisfactory for both instruments. It was slightly higher for the SCQ, which, similarly to the ADI-R, is completed by the parent/caregiver of the assessed individual. The SCQ was designed as a companion screening measure for the ADI-R [36], and the convergence of their results has been established empirically [54]. The slightly lower agreement between the ADI-R-PL and the ADOS-2 scores, though higher than the agreement (κ = 0.53) obtained by Lord et al. [4], may result from the fact that information in these two instruments is collected from different sources: in the ADOS-2 the assessment is made by a diagnostician who observes the person being evaluated. Furthermore, it does not take into account early development, only measuring his or her present functioning. The results support previous findings that data from the ADI-R and the ADOS-2 make independent contributions to ASD diagnoses [4].

### Algorithms for Toddlers and Young Preschoolers

The algorithms for toddlers demonstrated excellent interrater reliability. Intraclass correlation coefficients for test–retest reliability were excellent or satisfactory in most domains. The lowest score stability value was obtained for the Total Algorithm Score in the PH21–47 algorithm. Poorer stability over time in the case of toddlers is probably related to developmental dynamics [16], which likely affected the findings in our study as well.

Between-groups comparisons yielded statistically significant differences between the ASD group and control group, confirming that the new algorithms have good discriminant validity. The only exception was the Reciprocal and Peer Interactions (RPI) domain from the PH21–47 algorithm, in contrast to the results presented by Kim and Lord [16] and de Bildt et al. [15], where the domain scores were significantly higher for the ASD sample than the nonspectrum or typically developing groups. The RPI domain contains three items and assesses behaviors such as taking an interest in other children, the child’s responses to other children initiating contact, and the appropriateness of his or her social responses. A lack of statistically significant differences for RPI domain in the ADI-R-PL may have resulted from the size of the sample along with the young age of assessed children. At the preschool age children are developing their social skills and learning how to interact and play with their peers. However, the study sample was small and more data is necessary to determine the properties of the Reciprocal and Peer Interactions domain in the ADI-R-PL with greater accuracy.

Our data indicate better specificity of the algorithms for toddlers and young preschoolers than sensitivity, especially for the PH21–47 algorithm. We decided to use the original cutoff scores (which are different for clinical and research use; [16]) due to the small sample size. The specificity of the algorithms for toddlers in our sample (88% and 100% for the SW21–47 algorithm and 93% and 100% for the PH21–47 algorithm for the clinical and research cutoffs, respectively) was comparable to (or even higher than) results obtained by Kim et al. [17] ranging from 58 to 92% for SW21–47 and from 70 to 94% for PH21–47, depending on the dataset and cutoff. The sensitivity of the SW21–47 algorithm was adequate, much better for the clinical (92%) than the research cutoff (67%), and comparable to results obtained by Kim et al. [17]. Unfortunately, the sensitivity of the PH21–47 algorithm was quite poor, regardless of the cutoff (40%), and much lower than the sensitivity reported by Kim et al. [17], which ranged from 67 to 89%.

Our research has generated preliminary information about the psychometric properties of the new algorithms for toddlers and young preschoolers. The algorithms demonstrated robust reliability in the Polish version. They were also effective at differentiating the ASD group from non-ASD disorders and typically developing children. The sensitivities and specificities of the new algorithms indicated good diagnostic validity, especially for the clinical cutoff, with the exception of the poor specificity of the PH21–47 algorithm. Due to the size of the study sample, our results constitute only an initial stage of analysis of the properties of the ADI-R-PL. Moreover, the study enrolled children of at least 30 months of age, thus precluding any assessment of the usefulness of the ADI-R-PL in diagnosing the youngest children using the new algorithms. Further research in a larger sample that includes the youngest children, especially nonverbal aged 12–20 months, is therefore necessary.

### Strengths and Limitations

Our findings indicate that the ADI-R-PL demonstrates high reliability and validity. It should be noted that this is the first empirical research reporting separate cutoffs in three of the ADI-R domains for a non-English-speaking population. The analyses included three ways of estimating the ADI-R-PL’s reliability (interrater, test–retest, and internal consistency reliability) and several types of validity (diagnostic, discriminant, and convergent). Psychometric robustness, including high sensitivities, specificities, and positive predictive values, support the use of this instrument both in individual clinical diagnostics of ASD and in scientific research.

One limitation of the study was the size of the validation (*N* = 125) and normalization (*N* = 178) groups. The same was true of some other studies on the psychometric properties of non-English versions of the ADI-R [e.g. 27, 33, 34]. In particular, the small number of nonverbal participants prompted us to specify a common cutoff for verbal and nonverbal individuals in the Qualitative Abnormalities in the Communication domain of the Polish version of the ADI-R. The fact that there is no separate cutoff for nonverbal patients naturally limits the diagnostic usefulness of the instrument. Expanding the pool of data on nonverbal individuals is one of the key future goals associated with increasing the value of the ADI-R-PL.

In the case of older children and adults diagnosed with ASD, the time from ASD diagnosis to ADI-R-PL assessment was relatively long—more than one year, and up to several years for some older participants. In such circumstances the results of the ADI-R-PL may have been affected by the possible guardians’ difficulty in recollecting detailed information about the early development of the individual with ASD.

Another weakness was that the structure of the sample differed from the demographic structure of the general population in Poland, especially with respect to place of residence, since the majority of participants lived in large cities. Inhabitants of rural areas made up only 5.7% of the sample, while the proportion of rural residents in the Polish population is over 39% [55].

## Conclusions

Despite the limitations described above, the study is the first of its kind to conduct research on the Polish version of the ADI-R, especially with regard to the new algorithms for toddlers and young preschoolers, thus expanding our knowledge about the instrument. Importantly, it gives professionals in Poland access to the first standardized interview protocol for ASD, with verified psychometric properties. This is the first empirical research reporting novel cutoffs in three of the ADI-R domains for a non-English-speaking population, which we recommend using when assessing Polish participants. The study has generated preliminary information about the psychometric properties of the new algorithms for toddlers and young preschoolers. However, at this point, the new algorithms should not be used for clinical purposes and further research in a larger sample that includes the youngest children is necessary.

## Summary

This paper presents findings from the validation of the Polish version of the Autism Diagnostic Interview-Revised (ADI-R, [14]), including the original diagnostic algorithms and the new algorithms for toddlers and preschoolers. ADI-R is one of the most widely used standardized diagnostic instruments for autism spectrum disorder (ASD).

The study of reliability and validity of the ADI-R-PL included 125 individuals: 65 with clinical diagnosis of ASD (the ASD group) and 60 referred to collectively as the control group, with nonspectrum disorders and typical development. The normalization group was made up of 178 participants, including 118 with ASD and 60 controls.

The ADI-R-PL was found to have high interrater reliability, internal consistency, and test–retest reliability. The evidence of good diagnostic validity included high sensitivity (range 0.86–0.94), specificity (range 0.85–0.87), and positive predictive values (0.91–0.93) for the original diagnostic ADI-R-PL algorithm domains. Our data indicate high specificity of the algorithms for toddlers and young preschoolers. Unfortunately, the sensitivity of the PH21–47 algorithm was quite poor.

Between-groups comparisons revealed statistically significant differences between the ASD and control groups in all scales, subscales, and items making up the ADI-R algorithms, indicating solid discriminant validity of the instrument. The only exception was the Reciprocal and Peer Interactions domain from the new PH21–47 algorithm.

Good convergent validity of the instrument was confirmed by correlations of diagnoses made using the ADI-R-PL and those obtained with the Autism Diagnostic Observation Schedule, Second Edition, and Social Communication Questionnaire.

The results of the present study provide new information on a non-English version of the ADI-R, expanding our knowledge about the instrument. Preliminary analyses on the new toddler algorithms require further elaboration. At this point, the new algorithms should not be used for clinical purposes as opposed to the ADI-R-PL original algorithms for children aged 2 years or older, which can be applied in both clinical and research settings. To the best of our knowledge, this paper is the first to propose cutoffs in three ADI-R domains for a non-English-speaking population according to standards adopted in cross-cultural adaptation of assessment instruments.
